# Pre-transplant platelet-to- lymphocyte ratio predicts outcome after allogeneic hematopoietic stem cell transplantation

**DOI:** 10.1038/s41598-022-23344-0

**Published:** 2022-11-08

**Authors:** P. Woelfinger, B. Hauptrock, O. Kriege, A. List, T. Schmitt, R. Kuchen, M. Theobald, E. M. Wagner-Drouet

**Affiliations:** 1 Department of Hematology, Oncology and Pneumology, University Cancer Center Mainz (UCT), University Medical Center Mainz, Langenbeckstraße 1, Building 605 and 302, 55131 Mainz, Germany; 2 Institute for Medical Biostatistics, Epidemiology and Informatics (IMBEI), University Medical Center Mainz, Obere Zahlbacher Str. 69, Building 902, 55101 Mainz, Germany

**Keywords:** Cancer, Immunology, Stem cells, Biomarkers, Medical research, Oncology, Risk factors

## Abstract

For many patients with hematological malignancies such as acute leukemia or myelodysplastic syndrome allogeneic hematopoietic stem cell transplantation (allogeneic HSCT) is the only curative treatment option. Despite the curative potential of this treatment many patients experience relapse of their underlying disease or die due to multiple complications e.g. infections. Risk scores could help to assess the individual prognosis and guide patients and treating physicians to choose between different treatment options. Parameters reflecting the inflammatory status, such as neutrophil-to-lymphocyte ratio (NLR), monocyte-to-lymphocyte ratio (MLR), and platelet-to-lymphocyte ratio (PLR), have been demonstrated to be associated with prognosis and treatment complications in patients with various cancers. In this study, we evaluate pre-HSCT NLR, MLR and PLR as predictive markers in patients undergoing allogeneic HSCT. We demonstrate that a high (> 133) PLR level is associated with better clinical outcome. Patients with high pre-HSCT PLR show a significant better overall survival (*p* = 0.001), less relapses (*p* = 0.016), lower non-relapse-mortality (*p* = 0.022), less transfusions of red blood cells, platelets and fresh frozen plasma (*p* = 0.000), fewer episodes of fever (*p* = 0.002), considerably less different antibiotics (*p* = 0.005), fewer intensive care unit treatment (*p* = 0.017) and a lower in-hospital mortality (*p* = 0.024). Pre-HSCT PLR is easy to calculate by daily routine and could help to predict patient outcome after allogeneic HSCT.

## Introduction

For many hematological malignancies, such as leukemia or myelodysplastic syndrome, allogeneic hematopoietic stem cell transplantation (allogeneic HSCT) is the only curative therapeutic option^1,2^. Allogeneic HSCT is associated with severe treatment related complications such as infections, high transfusion need and graft versus host disease. Pre-transplant risk scores could be helpful to guide patients and treating physicians to choose the best individual therapeutic approach. Parameters reflecting inflammation, as for instance Neutrophil-to-Lymphocyte Ratio (NLR), Monocyte-to-Lymphocyte Ratio (MLR) and Platelet-to-Lymphocyte Ratio (PLR), are associated with prognosis and treatment complications in patients with various cancers, e.g. multiple myeloma^3–5^, lymphoma^5^, colon cancer^6^. They are also used in solid organ transplantation such as heart transplantation^7^ or liver transplantation^8^, but data in hematological diseases, especially in combination with allogeneic HSCT are rare or missing. In this retrospective study we evaluated the impact of NLR, MLR and PLR pre-HSCT (pre-HSCT NLR, pre-HSCT-MLR and pre-HSCT-PLR) on patient’s outcome after allogeneic HSCT.

## Patients and methods

### Patients

214 adult patients undergoing their first allogeneic HSCT at the Department of Hematology, Oncology and Pneumology, University Cancer Center Mainz (UCT), University Medical Center Mainz, Germany between January 2014 and December 2016 for hematological malignancies were included in our study and analyzed retrospectively.

All patients were treated following local standard protocols and had a lifelong follow-up routine in our outpatient unit at the University Hospital Medical Center in Mainz. Data were collected from patient charts retrospectively. Allogeneic HSCT was performed according to EBMT and JACIE guidelines (https://www.ebmt.org/accreditation/jacie-standards). The study was conducted in accordance with Good Clinical Practice Guidelines and the amended Declaration of Helsinki (1964). The study has been approved by the Landesaerztekammer Rhineland-Palatine Ethics Committee (Approval ID: 2018-13837) and their Institutional Review Board waived the need for informed consent.

123 patients (57.4%) were male and 91 (42.5%) female. Median age was 56.7 years (range 18–75 years). 106 (49.5%) patients were treated with acute myeloid leukemia (AML), 25 (11.7%) patients with acute lymphocytic leukemia (ALL), 10 (4.7%) patients with multiple myeloma (MM), 30 (14%) patients with myelodysplastic syndrome (MDS), 19 (8.9%) patients with lymphoma, 17 (7.9%) patients with myeloproliferative neoplasia (MPN) and 7 (3.3%) patients with other diseases. HLA-matched donors (10/10) were available for 146 patients (68.2%), 61 patients (28.5%) received transplants from 9/10 HLA-matched donors and 7 patients (3.3%) from 8/10 match donors.

29 (13.6%) patients received myeloablative conditioning regimen (MAC), 29 (13.6%) patients were treated with sequential conditioning regimen and 156 (72.9%) patients received non-myeloablative/reduced intensity conditioning regimen (RIC). Immunosuppressive therapy and conditioning regimen for all patients after allogeneic HSCT were diverse: 63 patients received alemtuzumab, fludarabine, melphalan (including Cyclosporine A), 29 patients received fludarabine, BCNU, melphalan (GVHD prophylaxis: Cyclosporine A and mycophenolate mofetil), 47 patients received fludarabine, busulfan, ATG (including Cyclosporine A and MTX). 29 patients with refractory AML were treated with sequential conditioning regimen (amsacrine, fludarabine, cytarabine, total body irradiation with 4 Gy and cyclophosphamide) combined with antithymocyte globulin, Cyclosporine A and mycophenolate mofetil). The other 44 patients were treated with different conditioning regimen.

Patient demographic and clinical characteristics are summarized in Table 1.

**Table 1:** Patient demographic and clinical characteristics.

Characteristic		All	NLR < 2.35	NLR > 2.35	p value	MLR < 0.31	MLR > 0.31	p value	PLR < 133	PLR > 133	p value
Total patients	n (%)	214 (100)	107 (50)	107 (50)	1	107 (50)	107 (50)	1	107 (50)	107 (50)	1
Age	(years) Mean; Median; Range	54.5; 57; 18–75	55.4; 58; 20–75	53.5; 56; 18–74	0.17	55.1; 58; 25–75	53.8; 57; 18–74	0.41	55.0; 58; 20–75	53.9; 57; 18–74	0.29
Sex	(M; F) n (%)	123;91 (57.5;42.5)	66;41 (61.7;38.3)	57;50 (53.3;46.7)	0.21	60;47 (48.8;51.6)	63;44 (51.2;48.4)	0.67	65; 42 (52.8; 46.2)	58; 49 (47.2; 53.8)	0.33
Disease	n (%)										
	AML	106 (49.5)	55 (51.9)	51 (48.1)		48 (45.3)	58 (54.7)		55 (51.9)	51 (48.1)	
	ALL	25 (11.7)	6 (24)	19 (76)		9 (36)	16 (64)		10 (40)	15 (60)	
	MM	10 (4.7)	4 (40)	6 (60)	0.054	4 (40)	6 (60)	0.044	1 (10)	9 (90)	0.094
	MDS	30 (14.0)	21 (70)	9 (30)		22 (73.3)	8 (26.7)		19 (63.3)	11 (36.7)	
	Lymphoma	19 (8.9)	10 (52.6)	9 (47.4)		9 (47.4)	10 (52.6)		8 (42.1)	11 (57.9)	
	MPN	17 (7.9)	8 (47.1)	9 (52.9)		12 (70.6)	5 (29.4)		10 (58.8)	7 (41.2)	
	Others	7 (3.3)	3 (42.9)	4 (57.1)		3 (42.9)	4 (57.1)		4 (57.1)	3 (42.9)	
HLA matching	n (%)										
	10;10	146 (68.2)	72 (49.3)	74 (50.7)		71 (48.6)	75 (51.4)		69 (47.3)	77 (52.7)	
	9;10	61 (28.5)	31 (50.8)	30 (49.2)	0.91	31 (50.8)	30 (49.2)	0.49	34 (55.7)	27 (44.3)	0.5
	8;10	7 (3.3)	4 (57.1)	3 (42.9)		5 (71.4)	2 (28.6)		4 (57.1)	3 (42.9)	
ECOG pre-HSCT	n (%)										
	0	180 (85.7)	84 (46.7)	96 (53.3)		88 (48.9)	92 (51.1)		86 (47.8)	94 (52.2)	
	1	25 (11.9)	16 (64)	9 (36)	0.23	13 (52)	12 (48)	0.85	16 (64)	9 (36)	0.28
	2	5 (2.4)	3 (60)	2 (40)		3 (60)	2 (40)		2 (40)	3 (60)	
CD34 + (× 106)/kg in the graft	Mean, Median, Range	6.15; 6.2; 1.1–9.4	6.1; 6.3; 1.1–9.4	6.1; 6.2; 2–8.9	0.65	6.2; 6.4; 2–9.3	6; 5.8; 1.1–9	0.24	6.1; 6.3; 2–9.4	6.1; 6; 1.1–9	0.63
Conditioning regimen	MAC n (%)	29 (13.6)	13 (44.8)	16 (55.2)		16 (55.2)	13 (44.8)		13 (44.8)	16 (55.2)	
	Sequential n (%)	29 (13.6)	19 (65.5)	10 (34.5)	0.18	12 (42.9)	16 (57.1)	0.63	18 (62.1)	11 (37.9)	0.34
	RIC n (%)	156 (72.9)	75 (48.1)	81 (51.9)		79 (50.6)	77 (49.4)		76 (48.7)	80 (51.3)	
GVHD prophylaxis	Alemtuzumab n (%)	65 (30.4)	36 (55.4)	29 (44.6)		38 (58.5)	27 (41.5)		37 (56.9)	28 (43.1)	
	ATG (%)	146 (68.2)	71 (48.6)	75 (51.4)	0.14	67 (46.2)	78 (53.8)	0.22	70 (47.9)	76 (52.1)	0.10
	Other n (%)	3 (1.4)	0 (0)	3 (100)		2 (66.7)	1 (33.3)		0 (0)	3 (100)	
HCT-CI	(Mean, Median, Range)	2.8, 3, 0–12	2.9, 3, 0–12	2.7, 3, 0–8	0.73	2.9, 3, 0–12	2.9, 3, 0–8	0.99	3.0, 3, 0–9	2.7, 3, 0–12	0.22
Duration of hospitalization	(days) Mean, Median, Range	43.6; 35; 12–195	44.6; 35; 12–171	42.6; 35; 15–195	0.67	44; 35; 12–195	43.2; 35; 15–171	0.85	47.5; 35; 12–195	39.6; 35; 14–133	0.26
BMI pre-HSCT	Mean, Median, Range	26.2; 25.6; 18–42	25.9; 25.4; 19–39	26.5; 25.7; 18–42	0.46	26.4; 25.8; 19–42	26; 25.6; 18–39	0.68	26.4; 25.7; 19–42	26; 25.6; 18–39	0.61
Granulocyte reconstitution (> 0.5/nl)	reached (days post-HSCT) Mean, Median, Range	18.5; 18; 9–37	18.3; 17; 10–34	18.7; 18; 9–37	0.54	18; 17; 9–34	19; 18.5; 10–37	0.09	18.3; 17; 10–37	18.7; 18; 9–32	0.31
Transfusions during hospitalization	Red blood cell transfusion (n, Mean, Median, Range)	10.8; 8; 0–100	12.2; 10; 0–100	9.3; 8; 0–50	0.06	10.6; 8; 0–50	11; 8; 0–100	0.57	13.1; 10; 0–50	8.3; 6; 0–100	0.000
	Platelet transfusion (n, Mean, Median, Range)	11.6; 8; 0–115	13.3; 9; 0–115	9.8; 6; 0–79	0.03	11.1; 8; 0–79	12; 7; 0–115	0.83	14.5; 10.5; 0–79	8.5; 6; 0–115	0.000
	FFP (n, Mean, Median, Range)	0.8; 0; 0–34	0.7; 0; 0–10	0.9; 0; 0–34	0.27	0.58; 0; 0–10	1; 0; 0–34	0.43	1.36; 0; 0–34	0.27; 0; 0–8	0.000
Fever during hospitalization	Days	3.2; 2.0; 0–26	3.6; 2; 0–26	2.8; 2; 0–14	0.22	3.5; 2; 0–26	2.9; 2; 0–14	0.35	3.8; 2; 0–26	2.61; 2; 0–15	0.002
Different antibiotics during hospitalization	Mean, Median, Range	1.9; 2.0; 0–6	2; 2; 0–5	1.7; 1; 0–6	0.05	2; 2; 0–6	1.8; 1; 0–6	0.22	2.1; 2; 0–6	1.6; 1; 0–6	0.005
ICU treatment during hospitalization	n (%)	42 (19.7)	22 (52.4)	20 (47.6)	0.7	24 (57.1)	18 (42.9)	0.31	28 (66.7)	14 (33.3)	0.017
In hospital mortality	Alive n (%)	192 (89.7)	96 (50)	96 (50)	1	96 (50)	96 (50)	1	91 (47.4)	101 (52.6)	0.024
	Dead (%)	22 (10.3)	11 (50)	11 (50)		11 (50)	11 (50)		16 (72.7)	6 (27.3)	
CMV reactivation	n (%)	90 (42.1)	52 (57.8)	38 (42.2)	0.053	52 (57.8)	38 (42.2)	0.053	47 (52.2)	43 (47.8)	0.58
EBV reactivation	n (%)	49 (22.9)	25 (51)	24 (49)	0.87	22 (44.9)	27 (55.1)	0.41	21 (42.9)	28 (57.1)	0.25
Acute graft versus host disease	Acute GVHD grade 1–4 n (%)	96 (45.9)	48 (50)	48 (50)	0.94	53 (55.2)	43 (44.8)	0.14	48 (50)	48 (50)	0.84
	Acute GVHD grade 3–4 n (%)	37 (17.7)	16 (43.2)	21 (56.8)	0.34	21 (56.8)	16 (43.2)	0.34	17 (45.9)	20 (54.1)	0.52
C-reactive Protein (CRP) pre-HSCT	Mean, Median, Range (mg/l)	17.5, 5.2, 0–259	21.8; 5.3; 0–259	13.2; 5.2; 0–138	0.58	21.5; 6.5; 0–259	13.5; 4.6; 0–138	0.37	21.6; 6.3; 0–259	13.4; 4.8; 0–138	0.068
Ferritin pre-HSCT	Mean, Median, Range (ng/ml)	1828, 1099, 12–10,663	2084; 1229; 31–10,663	1558; 1066; 12–10,002	0.15	1736; 912; 42–10,663	1367; 1259; 12–10,002	0.57	2294; 1477; 140–10,663	1337; 754; 12–10,002	0.004
	GVHD n (%)	7 (5.4)	3 (42.9)	4 (57.1)		3 (42.9)	4 (57.1)		6 (85.7)	1 (14.3)	
Causes of death	Relapse n (%)	53 (40.8)	25 (47.2)	28 (52.8)	0.76	20 (38.5)	32 (61.5)	0.072	28 (52.8)	25 (47.2)	0.34
	Sepsis n (%)	55 (42.3)	31 (56.4)	24 (43.6)		35 (63.6)	20 (36.4)		31 (56.4)	24 (43.6)	
	Other n (%)	15 (11.5)	8 (53.3)	7 (46.7)		8 (53.3)	7 (46.7)		10 (66.7)	5 (33.3)	

Blood values from 46 healthy donors served as controls (ctrl).

### Data availability

The data that support the findings of this study are available from the corresponding author, PW, upon reasonable request.

### Pre-transplant prognostic scores and definitions

Blood values obtained within 5 days previous to conditioning were used to calculate the pre-HSCT scores:

Pre-HSCT-NLR was defined as ratio between absolute neutrophil count and absolute lymphocyte count, pre-HSCT-PLR as ratio between absolute platelet count and absolute lymphocyte count, MLR as ratio between absolute monocyte count and absolute lymphocyte count as described in other indications^3,4,8^.

The median of each score was used as cut off: NLR (= 2.35), MLR (= 0.31) and PLR (133). We divided patients in 2 groups based on these cutoff values for further analysis: NLR > 2.35 (NLR high group) or NLR < 2.35 (NLR low group), MLR > 0.31 (MLR high group) or MLR < 0.31 (MLR low group) and PLR > 133 (PLR high group) or PLR < 133 (PLR low group).

Overall survival (OS) was defined as time from the date of allogeneic HSCT to date of death. Non relapse mortality (NRM) described death of every reason while in remission. Neutrophil engraftment was specified as the first of 3 consecutive days with absolute neutrophil count > 0.5/nl.

Based on the NLR, MLR and PLR high group and NLR, MLR and PLR low group, we compared both groups regarding OS, NRM and relapse within 1500 days after allogeneic HSCT.

### Statistical analysis

Categorical variables were depicted as numbers and percentages, continuous variables as means, medians and ranges. Correlations between the NLR, MLR or PLR groups (high or low group) were evaluated using chi-square test, Mann–Whitney-U test or Kruskal–Wallis test. In the case of OS, survival curves are obtained with the non-parametric Kaplan–Meier method. In the case of the two competing risks relapse and NRM, cumulative incidence curves (CIC) are used instead, since they provide a more practical interpretation with regard to treatment utility. The impact of studied variables was assessed by a multivariate Cox regression model for OS, relapse and NRM. All statistical analyses were performed using Graph Pad Prism 9.1.0. software (Graphpad Software Inc., USA, www.graphpad.com) and SPSS Version 26 (IBM, USA, www.ibm.com/de-de/analytics/spss-statistics-software). All p-values were two-sided, and *p* < 0.05 was considered statistically significant.

## Results

**Figure 1:**
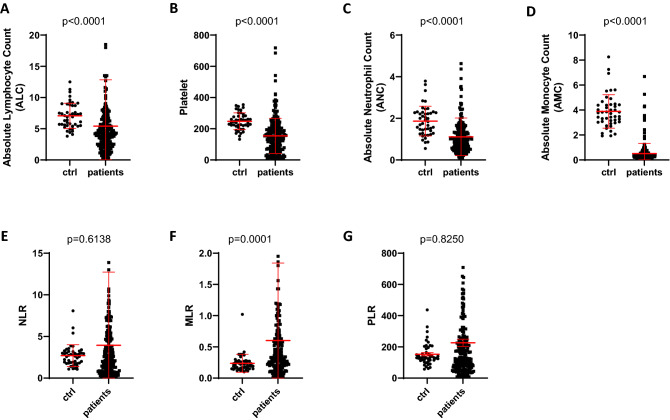
Comparison of ALC, Platelet, ANC and AMC count between healthy individuals (ctrl) and HSCT patients and comparison of NLR, MLR and PLR between healthy individuals (ctrl) and HSCT patients. HSCT Patients showed lower ALC (**A**), Platelet (**B**), ANC (**C**) and AMC (**D**) count compared to healthy individuals (ctrl). NLR (**E**), MLR (**F**) and PLR (**G**) was higher in HSCT patients compared to healthy individuals (ctrl).

A total of 214 adult patients with hematological diseases undergoing their first allogeneic HSCT were included in this retrospective analysis. All patients were observed up to 1500 days after allogeneic HSCT. First we compared all 4 blood count components of NLR, MLR and PLR (absolute monocyte count (/nl), absolute neutrophile count (/nl), absolute lymphocyte count (/nl) and absolute thrombocyte count (/nl)) between healthy individuals and our patients. Healthy individuals showed statistically significantly higher values in all parameters: absolute lymphocyte count (mean 7.7/nl vs. 5.4/nl; *p* < 0.0001, Fig. 1A), platelet count (mean 247/nl vs. 154/nl; *p* < 0.0001, Fig. 1B), absolute neutrophil count (mean 1.8/nl vs. 1.1/nl; *p* < 0.0001, Fig. 1C) and absolute monocyte count (mean 3.8/nl vs. 0.5/nl; < 0.0001, Fig. 1D). NLR (median patients 2.35 vs. healthy individuals 2.68, *p* = 0.61, Fig. 1E), MLR (median patients 0.31 vs. healthy individuals 0.20, *p* = 0.0001, Fig. 1F) and PLR (median patients 133 vs. healthy individuals 132, *p* = 0.82, Fig. 1G) were higher in patients compared to healthy controls, statistical significance could only be demonstrated for MLR.

**Figure 2:**
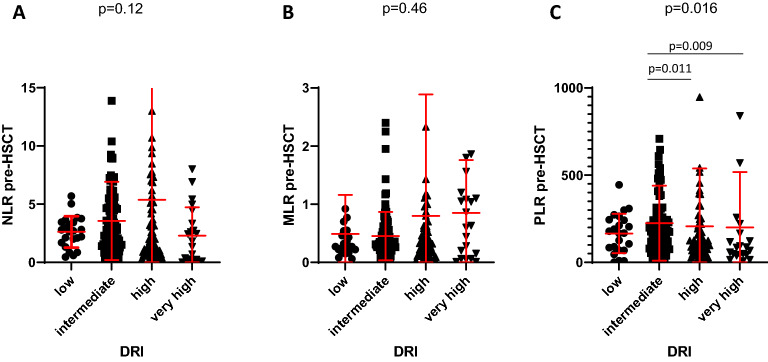
Association between NLR, MLR, PLR and Disease Risk Index (DRI). Regarding DRI no significant differences could be observed for NLR (*p* = 0.12, **A**) and MLR (*p* = 0.46, **B**). For PLR patients in the intermediate risk group showed significant higher PLR compared to patients in the high risk group (*p* = 0.009) and very high risk group (*p* = 0.011, **C**).

According to their pre-HSCT disease risk index (DRI)^9^ patients were distributed into 4 risk groups: Low risk (n = 23 (10.7%)), intermediate risk (n = 99 (46.2%)), high risk (n = 65 (30.3%)), very high risk (n = 21 (9.8%)), while 6 (2.8%) patients could not be assigned due to missing data. There were no significant differences in DRI risk groups with concern to NLR or MLR. Only for PLR significant differences could be observed (*p* = 0.0016): Patients in the intermediate DRI group showed significant higher PLR compared to patients in the high DRL group (*p* = 0.009) or very high DRI group (*p* = 0.011) (Fig. 2). Analyzing different types of conditioning regimen (MAC vs. sequential including patients with refractory AML vs. RIC) no significant differences were detected between NLR (*p* = 0.18), MLR (*p* = 0.63) or PLR (*p* = 0.34). With regard to different diseases, significant differences became apparent for MLR (*p* = 0.044), but not for NLR (*p* = 0.054) or PLR (*p* = 0.094) (Table 1).

Considering age, sex, HLA matching, ECOG, body mass index (BMI) pre-HSCT, CD34 + cells (× 10^6^)/kg in the graft, GVHD prophylaxis (Alemtuzumab vs. ATG vs. other), HCT-CI score, no statistically significant differences between NLR, MLR, PLR high groups and NLR, MLR and PLR low groups could be detected in univariate analysis.

We observed significant differences in our new defined groups when we investigated clinical outcome parameter:

Patients of PLR high group needed less red blood cell transfusions (mean 6; median 8.3; range 0–100 vs. mean 13.1; median 10.0; range 0–50; *p* = 0.000), less platelet transfusions (mean 8.5; median 6.0; range 0–115 vs. mean 14.5; median 10.5; range 0–79; *p* = 0.000) and less fresh frozen plasma (mean 0.27; median 0; range 0–8 vs. mean 1.36; median 0; range 0–34; *p* = 0.000). Analyses of infection and ICU treatment showed statistically significant differences as well: Patients within PLR high group had less episodes of fever > 38.5 °C (days: mean 2.61; median 2.0; range 0–15 vs. mean 3.8; median 2.0; range 0–26; *p* = 0.002) and received fewer different antibiotics (mean 1.6; median 1.0; range 0–6 vs. mean 2.1; median 2.0; range 0–6; *p* = 0.005). 42 patients were submitted to ICU, 14 (33.3%) of these were assigned to PLR high group compared to 28 (66.7%) patients matched to PLR low group (*p* = 0.017). 22 (10.3%) of 214 patients died during the hospitalization, 6 (27.3%) within PLR high group and 16 (72.7%) patients within PLR low group (*p* = 0.024). The PLR low group showed significantly higher pre-HSCT Ferritin levels (mean 2294; median 1477; range 140–10,663 vs. mean 1337; median 754; range 12–10,000 ng/ml; *p* = 0.004). Between NLR high group and NLR low group, significant differences could be observed regarding platelet transfusion (mean: 9.8; median: 6; range: 0–79 vs. mean: 13.3; median: 9; range: 0–115; *p* = 0.03) (see Table 1).

NLR, MLR and PLR did not result in differences regarding duration of hospitalization, time to granulocyte reconstitution (> 0.5/nl), development of acute Graft versus Host disease (aGVHD), Cytomegalovirus reactivation, Epstein-Barr-Virus reactivation post-HSCT or C-reactive Protein (CRP) pre-HSCT (Table 1).

130 (60.7%) patients died within the observation period of 1500 days after allogeneic HSCT, 63 (29.4%) had a relapse of the underlying disease, while 73 (34.1%) patients died in remission. Overall survival 1500 days after allogeneic HSCT was 39.2% (84/214) and median survival was 543 days. The 1500-day OS was significantly higher in the PLR high group (52/107 (48.5%) vs. 33/107 (30.8%), *p* = 0.001). Also 1-year-OS (75/107 (70.0%) vs. 51/107 (47.6%); *p* = 0.0005) and 2-year-OS (62/107 (57.9%) vs. 36/107 (33.6%); *p* = 0.0001) were significant higher in the PLR high group. No significant differences were observed between the NLR and MLR groups regarding OS (Fig. 3A, B and C day 1500 post-HSCT OS).

**Figure 3:**
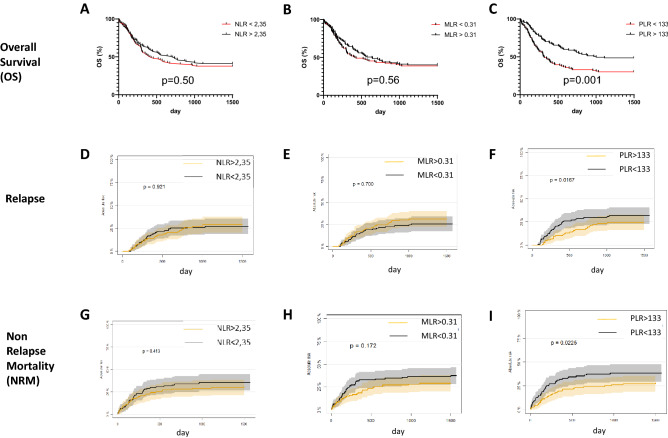
Association between NLR, MLR, PLR and Overall Survival (OS), Relapse as well as Non Relapse Mortality (NRM). (**A**), (**B**) and (**C**) show the association between OS (observation time 1500 days (= 4.1 years) post allogeneic HSCT) for NLR, MLR and PLR, while patients with PLR > 133 showed significant higher OS in comparison to patients with PLR < 133 (*p* = 0.001). (**D**), (**E**) and (**F**) show the association between the 3 markers and relapse, while patients with PLR > 133 showed significant lower relapse in comparison to patients with PLR < 133 (*p* = 0.016). (**G**), (**H**) and (**I**) show the association between NLR, MLR and PLR and NRM, while again patients with PLR > 133 showed significant lower NRM in comparison to patients with PLR < 133 (*p* = 0.02).

We observed a significant lower relapse incidence in the PLR high group 1500 days after allogeneic HSCT (*p* = 0.016). No significant differences could be detected at any other time point for NLR, MLR or PLR (Fig. 3D, E and F day 1500 post-HSCT relapse).

NRM was significant higher in the PLR low group after 1500 days: 44/107 (41.1%) vs. 29/107 (27.1%); *p* = 0.022). No significant differences were observed using the NLR or MLR score (Fig. 3G, H, I day 1500 NRM).

Additionally, we analyzed the causes of death focusing on GVHD, relapse, sepsis or other reasons. Yet, no significant differences were observed (see Table 1).

In a multivariate analysis focusing on OS, relapse and NRM adjusted for NLR, MLR, PLR, DRI, conditioning regimen, GVHD prophylaxis, CD34 cells/kg, HCT-CI and age, the PLR was found to be an independent prognostic factor for OS (hazard ratio [HR] 0.56, *p* = 0.009). Three more independent prognostic factors for OS were GVHD prophylaxis, HCT-CI, conditioning regimen and age for NRM (Table 2).

**Table 2:** Multivariate analysis for OS, Relapse and NRM.

	OS	Relapse	NRM
	HR (95% CI) *p* value	HR (95% CI) *p* value	HR (95% CI) *p* value
NLR (< 2.35 vs. > 2.35)	1.33 (0.85–2.06) 0.20	0.85 (0.46–1.56) 0.6	1.27 (0.69–2.35) 0.42
MLR (< 0.31 vs. > 0.31)	0.98 (0.65–1.47) 0.93	1.51 (0.86–2.66) 0.14	0.8 (0.45–1.43) 0.46
PLR (< 133 vs. > 133)	0.56 (0.36–0.86) 0.009	0.83 (0.47–1.47) 0.53	0.61 (0.33–1.12) 0.11
DRI (low/intermediate vs. high/very high)	0.9 (0.59–1.35) 0.61	1.05 (0.61–1.81) 0.84	0.74 (0.42–1.33) 0.32
Conditioning regimen (MAC/Sequential vs. RIC)	0.53 (0.31–0.89) 0.017	0.58 (0.3–1.1) 0.09	0.56 (0.26–1.19) 0.13
GVHD prophylaxis (Alemtuzumab vs. ATG)	0.58 (0.37–0.90) 0.016	0.69 (0.37–1.29) 0.25	0.59 (0.33–1.08) 0.08
Age (< 56.7 years vs. > 56.7 years)	1.35 (0.89–2.04) 0.15	0.74 (0.43–1.26) 0.27	1.95 (1.08–3.53) 0.02
CD34 cells/kg (< 6.2 vs. > 6.2)	1.28 (0.89–1.85) 0.17	0.88 (0.54–1.45) 0.63	1.4 (0.83–2.36) 0.2
HCT-CI (1–3 vs. 4–12)	1.52 (1.04–2.21) 0.028	1.44 (0.86–2.41) 0.15	1.34 (0.79–2.27) 0.27

## Discussion

For many hematological diseases, allogeneic HSCT is the only curative therapeutic option^1,2^. Yet, this therapy is associated with high treatment complications, morbidity and mortality^10–13^. Several criteria were introduced in clinical practice to predict outcome after allogeneic HSCT to select and advise patients. Recently used scores were EASIX score, HCT-CI score or the EBMT score to predict outcome after allogeneic HSCT^14–17^. Most of these criteria are based on disease status, comorbidities or other pre-HSCT parameters. Recently, parameters reflecting the inflammatory status such as NLR, MLR and PLR have been recognized to play an important role to identify patients with higher risk of NRM, relapse and impaired OS especially in solid cancers or solid organ transplantation settings^6,7,18–21^. Data in hematological diseases, especially in autologous or allogeneic stem cell transplantation are rare or missing. A growing number of studies support the use of a combination of various acute phase proteins to develop composite, inflammation-based prognostic scores, which include the NLR, MLR and PLR. Recent literature gives an overview of different types of cancer and solid organ transplantation in which NLR, MLR and PLR are able to predict outcome in different settings: In patients after heart transplantation, low NLR and low PLR were associated with better cumulative survival^7^. Lower NLR predicted better OS in patients with colon cancer^18^. Regarding hematological diseases, data are rare: No data were available regarding NLR or MLR and different types of newly diagnosed acute leukemia or allogeneic stem cell transplantation. One study reported that PLR did not predict outcome in patients with chronic lymphatic leukemia^22^. In patients with diffuse large B-cell lymphoma, elevated PLR^23^ and elevated NLR^24^ were associated with poor prognosis. Elevated NLR and MLR and in contrast decreased PLR were associated with unfavorable clinicobiological features in multiple myeloma patients^4^. What is more, increased NLR, MLR, and PLR predicted poor clinical outcome in MM patients at day + 100 after autologous stem cell transplantation^3^. Stefaniuk et al. described a better prognosis in patients with different types of lymphoma or multiple myeloma and low NLR, but for CLL a high NLR was associated with a better prognosis. Stefaniuk mentioned only one study investigating patients with AML and no data regarding allogeneic HSCT^5^. In summary, the prognostic impact of NLR, MLR and PLR in hematological diseases are not consistent and no data are available for allogeneic HSCT settings. However, in most analysis, high and not low NLR, MLR and PLR are associated with worse outcome in different diseases and therapy strategies.

Our data investigating patients with haematologic malignancies treated with allogeneic HSCT are not completely consistent with previous studies in other clinical settings, which indicated that high NLR, MLR and PLR predict poor outcomes in different cancer settings. For the first time, we show that high and not low pre-HSCT PLR predict better OS, less relapses and lower NRM, while NLR and MLR failed to predict OS, relapse and NRM. Regarding other clinical parameters, especially patients with high PLR were transfused to a lesser extend, had fewer episodes of fever and needed less changes in antibiotics or ICU treatment. Furthermore, in the high pre-HSCT PLR group, in-hospital mortality was lower.

Patients with solid tumors mainly did not show any myelo suppression. In these patients, high scores dealing with inflammation markers values identify patients at risk of tumor progression as described above. In the setting of allogeneic HSCT or in hematological malignancies, such as leukemia or myelodysplastic syndrome, high platelet counts represents better bone marrow function, which could be due to less aggressive disease itself, less intense therapeutic treatment before transplantation or good response to the last treatment line. This could reflect a better disease specific prognosis. Therefore, high PLR pre-HSCT might not only reflect the inflammatory status, but also be a potential predictor of better bone marrow function itself, lower activity of the hematological malignancy, or less harmful pre-treatment, such as chemotherapy, radiation or autologous stem cell transplantation with less toxicity. From our consideration the association of high PLR with better clinical outcomes after allogeneic HSCT is consistent. These findings are in line with the observation in patients with lymphoma and multiple myeloma, but differ to publications based on solid malignancies and are characteristic for hematological diseases.

Nevertheless, our study has several limitations: First of all, this analysis is a retrospective, single-center study. Secondly, this study mainly dwelled on allogeneic HSCT in general, without focusing on the different underlying malignancies, conditioning regimen or other allogeneic HSCT-specific parameters that could lead to differences in the investigated scores. In addition to PLR, conditioning regimen and GVHD prophylaxis had also significant impact on OS using a multivariate Cox model (covariates age, CD34 cells/kg, HCT-CI, NLR, MLR and DRI).

The influence of the conditioning regimen is expected as the sequential conditioning regimen was used for patients with refractory AML with worse prognosis per se. Alemtuzumab based regimen were only applied in patients with complete remission, reflecting a better prognosis regarding to relapse. Hence, these two risk factors are strongly influenced by the disease state itself, and can thus not be regarded as independent by definition.

As there was no significant impact of DRI our risk scores, especially PLR, seem to reflect additional information and did not represent disease risk solely. Especially the association with clinical parameters as transfusion, fever and ICU treatment or in hospital mortality indicate a prognostic expressiveness independent from the disease risk.

In conclusion, our study suggests that particularly pre-HSCT PLR could be used as pre-HSCT prognostic factor for patients who undergo allogeneic stem cell transplantation**.** In the future, it seems to be beneficial to generate more specific data in general for different types of blood cancers and their specific therapy strategies, in order to gain more differentiated evidence for the predictive value of NLR, MLR and PLR.
